# A Case of Strangulated Hernia in a Man Aged 82 Years

**Published:** 1846-12

**Authors:** Jesse Harvey

**Affiliations:** Harveysburg, Ohio


ARTICLE II.

*A case of Strangulated Hernia in a man aged* 82 *years.* By Dr. Jesse Harvey, of Harveysburg, Ohio.

B. M., aged 82 years, was afflicted with inguinal hernia of the intestines for about eleven years; the bowel often passing down but returning again without difficulty. About the middle of the third month, 1844, it became so strangulated, that a physician, who was called in, returned it with considerable difficulty. About two weeks after this time, while exercising, the bowel passed entirely down into the scrotum, filling the sack completely. He went to bed and made the usual efforts to return it; but failing, sent to my office about 6 o'clock in the evening, for assistance. At about 8 o'clock I arrived at his house, found him in great pain, and immediately entered upon the regular routine of operations, by the taxis, &c., for his relief. After an hour's labor I felt that I should fail,—bled freely and tried again with no better success. I wished to try the tobacco injection, but believing it would require to be carried to the last extreme to succeed, I desired counsel. While he was being brought, I continued my efforts, but to no purpose. When the physician arrived we commenced again, and after an hour the case was pronounced a hopeless one, except relieved by an operation, the tobacco injection having been carried to full effect.

Now about 12 o'clock at night further counsel was requested, the physician previously called, declaring that his nerves would not permit him to witness the operation. A messenger was dispatched for my friend Dr. Fisher, of Waynesville, who did not arrive until about 9 o'clock the next morning. We then applied ice to the parts for an hour or two, and made such efforts as were thought best, but all failed, and at about 12 o'clock we commenced the operation.

The sack was carefully opened with the scalpel and the stricture of the ring divided sufficiently to allow the bowel to return; but adhesions had formed, that prevented. I passed my finger into the vaginal sack, broke up the adhesions between the two serous membranes, and the intestine was replaced. The wound was dressed and a dose of calomel, ipecac. and morphine, was given, to be followed by a portion of castor oil with spts. turpentine.

Called the next day,—the bowels had been freely moved. Repeated the prescription of yesterday. The diet to be toasted bread and water sweetened.

The next day, 4th month, 6th, same prescription continued.

7th, mercurial action apparent and the calomel discontinued —oil of turpentine given in small doses every fourth hour, until the bowels were moved—this was continued daily until the 10th. The patient improved rapidly, the wound healed kindly and on the 14th my visits were discontinued.

About a month after this, he suffered a severe cholic, which with difficulty was relieved. He recovered from it rapidly, and enjoys good health. The operation did not close the ring, yet with the aid of a truss, he can walk or ride even on horseback, and makes baskets for a livelihood.

The ice had at least the good effect to restrain hemorrhage as the quantity lost was not sufficient to be troublesome during the operation.

